# A mixed-method research to investigate the adoption of mobile devices and Web2.0 technologies among medical students and educators

**DOI:** 10.1186/s12911-016-0283-6

**Published:** 2016-04-19

**Authors:** Si Fan, Jan Radford, Debbie Fabian

**Affiliations:** Faculty of Education, University of Tasmania, Locked Bag 1307, Launceston, TAS 7250 Australia; Launceston Clinical School, University of Tasmania, Locked Bag 1377, Launceston, TAS 7250 Australia

**Keywords:** Mobile devices, Web 2.0 technologies, Medical education, Digital habitat, digiMe

## Abstract

**Background:**

The past decade has witnessed the increasing adoption of Web 2.0 technologies in medical education. Recently, the notion of digital habitats, Web 2.0 supported learning environments, has also come onto the scene. While there has been initial research on the use of digital habitats for educational purposes, very limited research has examined the adoption of digital habitats by medical students and educators on mobile devices.

This paper reports the Stage 1 findings of a two-staged study. The whole study aimed to develop and implement a personal digital habitat, namely digiMe, for medical students and educators at an Australian university. The first stage, however, examined the types of Web 2.0 tools and mobile devices that are being used by potential digiMe users, and reasons for their adoption.

**Methods:**

In this first stage of research, data were collected through a questionnaire and semi-structured interviews. Questionnaire data collected from 104 participants were analysed using the Predictive Analytics SoftWare (PASW). Frequencies, median and mean values were pursued. Kruskal Wallis tests were then performed to examine variations between views of different participant groups. Notes from the 6 interviews, together with responses to the open-ended section of the questionnaire, were analysed using the constructivist grounded theory approach, to generate key themes relevant to the adoption of Web 2.0 tools and mobile devices.

**Results:**

The findings reflected the wide use of mobile devices, including both smart phones and computing tablets, by medical students and educators for learning, teaching and professional development purposes. Among the 22 types of Web 2.0 tools investigated, less than half of these tools were frequently used by the participants, this reflects the mismatch between users’ desires and their actual practice. Age and occupation appeared to be the influential factors for their adoption. Easy access to information and improved communication are main purposes.

**Conclusions:**

This paper highlights the desire of medical students and educators for a more effective use of Web 2.0 technologies and mobile devices, and the observed mismatch between the desire and their actual practice. It also recognises the critical role of medical education institutions in facilitating this practice to respond to the mismatch.

**Electronic supplementary material:**

The online version of this article (doi:10.1186/s12911-016-0283-6) contains supplementary material, which is available to authorized users.

## Background

This project is an initiative that highlights the shift from traditional teaching methods to personalised virtual learning environments (PLEs) and mobile devices. Traditionally, the technology of choice used for teaching in university contexts focused primarily on learning management systems (LMSs). However, with the emergence of other freely available technologies, LMSs are becoming less compatible. The uniform teaching methods offered by LMSs can no longer cater for the diversity of students today [1, 2], due to the increasing learner backgrounds and demands in Australian universities. Individuals differ in their readiness and capacity to adopt technologies in response to learning and workplace challenges, even given similar environments and demands. With this in mind, the idea of a “one size fits all” teaching method is counter-intuitive. Also, these freely available applications, contents and services are providing students and academics with a more attractive look, feel and functionalities that cannot be offered by LMSs, and therefore, are becoming increasingly popular among these users [3].

This project was conducted in responding to these changes. It is built upon the notion of personalised virtual learning environments (PLEs) (e.g., [3–6]) and the idea of a “digital habitat”, both of which have received intensive discussions in the past decade. Typically, in PLEs, a range of tools, instead of an actual system containing selected tools, are made available to learners [3]. This notion is followed by Wenger, White, and Smith [7] who refer to a “digital habitat” as being a collaborative learning space which arises from engagement with Web 2.0 technologies. By allowing choices on the tools and technologies, PLEs and digital habitats provide a stronger potential to enable and support individualised learning, and therefore, lead to more flexibility for higher education.

### The medical education context

The particular context in which this study took place was the School of Medicine at one Australian university: the University of Tasmania. Medical students and educators are significantly time poor [8]. This characteristic is of paramount importance when contemplating learning and adopting new technologies. This also leads to the interest of these user groups in the adoption of Web 2.0 technologies. Examples of these technologies include podcasts, blogs, instant messaging, wikis, and social bookmarking. In the past, medical students and clinicians were aware of Web 2.0 technologies, and possessed a high interest in their use, but the adoption was low [9]. In the past 10 years, however, apps for mobile devices have come onto the scene and the use of mobile technologies by medical students and health professionals has significantly increased. In the school where this project was conducted, for instance, the medical students reported active use of mobile devices [10]. Web 2.0 tools were accessed daily by 81 % of the medical students using their smartphones [10]. This active adoption triggered the initiation of this project, which is to build and introduce a Web 2.0 supported digital habitat to facilitate personalised learning.

### Focus of this paper

This paper reports Stage 1 of the research. The larger scope study involved two stages: 1) Stage 1, which investigated the types of devices and Web 2.0 tools used by students and staff in the school and reasons for the adoption; and 2) Stage 2, in which a Web 2.0 supported digital habitat was developed, trialled and evaluated. This paper reports the findings of the first stage.

The Web 2.0 supported digital habitat built in this project was named digiMe, and was developed for undergraduate medical students and clinical and professional staff at this particular school. digiMe facilitates the integration of freely available Web 2.0 technologies of the user’s choice into their learning and work processes using mobile devices. In this research, digiMe performed as an awareness tool, a technology and networking access point, as well as a learning space.

The findings of the first stage, as introduced in this paper, are of particular importance, as they provided a user analysis for Stage 2 of the project. As mentioned in previous sections, this project highlights the shift from traditional learning management systems to more personalised virtual learning environments. At the same time, it also recognises the increasing need among users for being able to access these PLEs using mobile devices, such as tablet computers and smartphones. Therefore, to inform the digital habitat development in the second stage, Stage 1 of the research placed its focus on two main aspects: the types of devices used in medical education and the Web 2.0 tools adopted by this user group.

### Use of mobile devices and apps

Since 2009, tablet computer and smartphone use by health professionals has and continues to increase; therefore, it can be expected that a desire for such portable devices to fulfil this mobile computing role should likewise increase [10, 11]. A study conducted in 2011, at the school in which this project was conducted, found that mobile devices were used by 85 % of its medical students and clinicians, primarily to manage information needs, time allocation, and to communicate [12]. The research concluded that mobile devices in clinical settings would soon be ubiquitous as they have the capacity to enhance learning and patient care.

There have also been an increase in the adoption of apps and reference resources using mobile devices. For instance, Davies et al.’s [13] study, involving 387 third and fourth year medical students, identified that learning was enabled by using PDAs (Personal Digital Assistants) preloaded with reference resources. Another study, with responses from 257 medical students and 131 junior doctors, found that the majority of those surveyed owned 1 to 5 medical apps, with few owing more than 10 [14]. Further research classified the popularly used apps into the categories of patient care and monitoring, health apps for users outside of the health profession for promoting general wellness, apps that enhance communication, education and researching, as well as reference apps for physicians and medical students [15].

### Use of Web 2.0 tools

Compared to the use of mobile devices, Web 2.0 adoption in medical education has a longer history. Mobile apps enable learning regardless of geographic location or device used. As such sharing content in visually interesting ways, especially videos, images, and presentations, can easily be achieved because of their portability and ease of use [1]. Web 2.0 tools, on the other hand, allow users to individually or collectively create and share content in a transparent, open and collaborative manner as opposed to the confines of institutional technologies [16]. Discussions around Web 2.0 adoption include both strengths and challenges. While courses have been developed to incorporate Web 2.0 technologies, it is considered a challenge to balance communication gain with increase in distractions, known shortcomings, and errors [17].

Cheston, Flickinger and Chisolm’s [18] literature review found that Web 2.0 tools were popular with learners and there was potential for using it for professional development and collaboration. The shared process and content, and accessible and customisable nature of Web 2.0 technology presented opportunities for its use in medical education [18]. Facebook is use by medical students for communication purposes [17]. Twitter and other Web 2.0 tools are being used by physicians in the US to bring a dynamic approach to teaching and learning [19]. Opportunities for the use of Web 2.0 tools for healthcare organisations extended to connecting with the community with regards to fundraising, news and information, patient education, advertising new services and customer service [20]. Web 2.0 tools provide a new avenue in which “people interact, present ideas and information, and judge the quality of content and contributions” [1]. It is also believed that tacit knowledge and skill sharing amongst clinicians using Web 2.0 tools can have a significant impact on the quality of medical diagnosis and decision making [21].

## Methods

As stated in the previous section, this paper reports the first stage of a larger scope study. Stage 1, as reported in this paper, used both qualitative and quantitative data collection and analysis methods. It was conducted through an online questionnaire and semi-structured interviews. The online questionnaire was designed using the SurveyMonkey data collection website. Part A of the questionnaire asked about the participants’ demographic information (see Table 1). Questions in Part B, Part C and Part D are provided in Additional file 1. The link to the questionnaire generated by SurveyMonkey was then distributed to all medical students (N = approx. 90) and staff (N = approx. 30) in one of the clinical schools in the School of Medicine. All students in this clinical school were in their Year 4 or Year 5 of their Bachelor of Medicine degree. Information about the study was introduced to the students in two year group meetings (one for Year 4, and one for Year 5), and the students were given time after the meeting to complete the questionnaire on their chosen digital devices. As a result, there were 104 questionnaire responses including those from both students and staff, resulting a high response rate of 94 %. The data collected through the questionnaire were analysed using the Predictive Analytics SoftWare (PASW) [22]. Frequencies, median and mean values were pursued. Kruskal Wallis tests were also performed as a post hoc test on some of the items where statistical significance emerged between responses from the different participant groups.

**Table 1:** Participant demographic information

Participant groups	Questionnaire	Interview
	% (n/N)	n/N
Occupation		
• Medical student	75.0 % (78/104)	0/6
• Clinical teachers/supervisors	17.3 % (18/104)	2/6
• University of Tasmania administrative staff	2.9 % (3/104)	2/6
• Academics	1.9 % (2/104)	1/6
• Others (e.g., Researchers)	2.9 % (3/104)	1/6
Gender		
• Male	42.3 % (44/104)	1/6
• Female	57.7 % (60/104)	5/6
Age		
• 20–29	74.0 % (77/104)	0/6
• 30–39	4.8 % (5/104)	0/6
• Over	39 21.2 % (22/104)	6/6

In addition to the questionnaire, 6 face-to-face interviews were conducted. These were semi-structured interviews and were guided by a list of questions (see Additional file 2). The 6 interviewees were selected through purposive sampling from those who expressed their interest after the completion of the questionnaire. All of these interviewees were staff, as no student volunteered to participate in the interview. Notes from the interviews, together with the data collected from the open-ended section of the questionnaire, were analysed using the constructivist grounded theory approach [23], and using NVivo 10 software as a tool [24]. In particular, the three-step coding approach in the constructivist grounded theory approach was used to generate key themes from these textual data [23]. Table 1 outlines the occupation, gender and age of the questionnaire and interview participants.

## Results

### Types of devices and Web 2.0 tools used

Findings presented in this section were collected from the participant responses to Part B and Part C of the questionnaire. Among the 104 questionnaire participants, 49 were using only smartphones for learning and teaching or professional development activities, and 3 were using only computing tablets, with 34 of them using both types of devices. The types of smart phones used included iPhones (*n* = 58), Android smartphones (*n* = 26), and Blackberry smartphones (*n* = 2). None of these participants was using smartphone with a Windows system. The types of computing tablets, on the other hand, included iPad (*n* = 31), Android tablets (*n* = 3), Blackberry tablets (*n* = 2), and Windows tablets (*n* = 1). It is also worth to note that 14 of these participants were not using any of these mobile technologies. However, these participants would be using laptop or desktop computers to fulfil these learning and teaching or professional development purposes. These students and staff would not be disadvantaged, as currently the use of mobile devices is not part of the curricular expectations within the coursework or within the professional job expectations.

In relation to Web 2.0 technology adoption, the participants were asked to rate the frequency of usage on a scale of 1, representing “Never used”, to 4 representing “Used very frequently”. Some tools were more frequently used (Median = 3.00), such as SMS, social networking tools, and Learning Management Systems, including MyLO (My Learning Online) which is the learning system used at the University of Tasmania. The participants also gave a relatively more positive response to wikis (Median = 3.00), although it is suspected that some respondents may have considered this question in relation to using wikis for retrieving information from Wikipedia, whereas the intention was to determine the use of wikis for authoring purposes. Cloud storage, video/slide sharing, and slide creation tools were also used on a number of occasions (Median = 2.00). The other tools, such as Blogs and Social Bookmarking, were not used (Median = 1.00). Frequencies of usage of these tools are presented in Table 2 through frequencies and median values.

**Table 2:** Web 2.0 tools used by questionnaire participants

Types of Web 2.0 tools	Never	Occasionally	Frequently	Very frequently	Total number	Median
Instant messaging	11	13	28	46	98	3.00
Social networking tool (e.g., Yammer, Facebook)	25	9	22	41	97	3.00
Cloud storage (e.g., DropBox, iCloud)	31	31	14	20	96	2.00
Learning Management System (e.g., MyLO, Moodle)	14	13	35	35	97	3.00
Web authoring tool	83	9	0	2	94	1.00
Wiki	13	15	27	44	99	3.00
Blog	79	13	1	1	94	1.00
SmartBoard	77	12	3	3	95	1.00
eReader	60	15	10	9	94	1.00
Annotating tool (e.g., iAnnotate)	78	10	3	3	94	1.00
Social Bookmarking	85	6	2	1	94	1.00
Collaborative authoring tool (e.g., GoogleDocs)	64	26	2	3	95	1.00
Digital stylus for writing/drawing	86	5	3	1	95	1.00
Video capture tool	77	12	1	3	93	1.00
Video/slide viewing (e.g., YouTube)	18	40	22	15	95	2.00
Slide creation tool (e.g., PowerPoint, Slideshare)	18	48	22	9	97	2.00
Audio recording tool	69	19	3	4	95	1.00
Audio listening tools (podcasts)	60	23	5	7	95	1.00
Desktop capture (e.g., Echo 360, Camtasia)	84	6	0	2	92	1.00
Online chat (e.g., MSN)	64	19	4	8	95	1.00
Web conferencing (e.g., Elluminate)	77	15	5	1	95	1.00
VOIP internet tele- or video-conferencing (e.g., Skype)	54	39	4	3	100	1.00

Likert scale: 1 = Never; 2 = Occasionally (less than once per week); 3 = Frequently (1 to 5 times per week); 4 = Very frequently (6 or more times per week)

Kruskal Wallis tests were performed as a post hoc test, to find out whether statistical differences exist between the different occupation, gender and age groups. Some of these tests produced positive results. Statistical significant differences were found on 6 types of Web 2.0 tools by the occupation groups, 1 type of Web 2.0 tools by the gender group, and 13 types by the age groups (see Table 3). Therefore, it can be interpreted that age is the most influential factor on the participants’ Web 2.0 adoption. Those Kruskal Wallis test results that are statistical significant are presented in Table 3.

**Table 3:** Statistical significant results from Kruskal Wallis tests

Types of Web 2.0 tools	Occupation	Gender	Age
Social networking tool (e.g., Yammer, Facebook)	*p* = .000	-	*p* = .001
Learning Management System (e.g., MyLO, Moodle)	*p* = .000	-	*p* = .000
Web authoring tool	-	-	.035
Wiki	*p* = .000	*p* = .017	*p* = .000
SmartBoard	-	-	*p* = .007
eReader	*p* = .026	-	-
Social Bookmarking	-	-	*p* = .027
Digital stylus for writing/drawing	-	-	*p* = .000
Video capture tool	-	-	*p* = .000
Video/slide viewing (e.g., YouTube)	*p* = .003	-	*p* = .001
Slide creation tool (e.g., PowerPoint, Slideshare)	*p* = .044	-	-
Audio recording tool	-	-	*p* = .002
Audio listening tools (podcasts)	-	-	*p* = .012
Desktop capture (e.g., Echo 360, Camtasia)	-	-	*p* = .032
Web conferencing (e.g., Elluminate)	-	-	*p* = .008

Asymptotic Significances are displayed. The significance level is .05. Only results that are statistically significant (<.05) are displayed in this table

Regarding the differences between occupation groups, the medical student group tended to use social networking tools (mean rank = 51.68), learning management systems (mean rank = 55.46), wikis (mean rank = 59.24), and video/slide viewing tools (mean rank = 52.92) more often than the other groups. The academics (mean rank = 67.00) and admin staff involved (mean rank = 84.25), however, used slide creation tools more often than students (mean rank = 48.79) and clinical teachers/supervisors (mean rank = 34.65). In addition, gender differences are only found on the use of wikis. That is, males (mean rank = 57.25) enjoyed interacting and doing collaborative works through wikis more than females (mean rank = 44.20).

Regarding the differences between age groups, the participants between 20 and 29 years of age were significantly more active in using some of the tools, such as social networking tools (mean rank = 54.30), learning management systems (mean rank = 55.60), and wikis (mean rank = 58.27). The 30–39 age group is more engaged in using web authoring tools (mean rank = 66.38), smartboards (mean rank = 75.00), social bookmarking (mean rank = 66.75), digital stylus for writing/drawing (mean rank = 80.13), video capture tools (mean rank = 85.63), audio recording (mean rank = 81.75) and audio listening (mean rank = 80.50). The two groups “50 to 59” and “60 and over” showed the most usage among all age groups for audio recording tools and web conferencing tools. One reason could be that the majority of academics, and clinical teachers/supervisors, who have more access to these tools for working purposes, are within this age group.

### Purposes of digital device and Web 2.0 adoption

Findings presented in this section emerged from the open-ended section (Part D) of the questionnaire and the interview data. In particular, this section presents three dominant reasons that emerged from the data: 1) quick and easy access to information; 2) reliability, interoperability and connectivity; and 3) improved workflow and communication.

A dominant purpose for digital devices and Web 2.0 adoption is quick and easy access to information. The participants in this study expressed their desire to access information from any places using whatever devices that are appropriate or available. It is expected by these participants that increased access would help enhance both learning and clinical practice. Examples of this information include guidelines, drug information, e-texts, teaching materials, and current evidence-based information to support decision-making and treatments. Information that can be used for patient education and that is relevant to the Australian context was found to be particularly useful. A medical student stated:

*It is impossible to stay updated with all the changes in medical recommendations (e.g. first line treatments seem to change year-by-year. Apps such as the therapeutic guidelines can be put on mobile phones and iPads, etc., rather than students/doctors trying to memorise the therapeutic guidelines every year.*

Reliability, interoperability and connectivity were mentioned both as reasons for adoption and criteria used for tool selection. In this study, the participants emphasised the importance of these factors to enable integration of information sources and seamless transition between platforms, devices and systems. Trustworthiness was identified as a paramount descriptor with regards to information. Criteria being used to determine the trustworthiness include: the quality of apps being adopted, qualifications of people who recommend the information, and credibility of forums through which people communicate. As such, many participants recommended resources and tools to be curated by suitably qualified persons. A clinical supervisor expressed:

*[I] would like to be updated on [the] latest apps for Android/iPhone devices – have them available for download, a place to review them… If there was some way I could access quality sites without having to worry about whether the site aligns with current best practice this would be highly beneficial – perhaps a forum where people could share sites found to be beneficial in practice would be good.*

Improved workflow and communication emerged to be the third dominant reason for adoption. From an administrative and teaching perspective, it was felt that Web 2.0 technologies offered opportunities for improved workflow (e.g., student e-portfolios versus paper portfolios) and information flow to and from students. They were particularly useful as a means to communicate timetable changes which otherwise would present ongoing challenges. From a clinical perspective, use of digital devices and Web 2.0 tools, such as including chat, forums and videoconference, increase the potential for improved communication with other health professionals and patients. For instance, a clinical supervisor stated:

*…once you refer a patient, you don’t necessarily know if they went … the patient pathway, I can never know where that is up to … Improved communication with patients by having mobile technology would assist [with] explanations.*

### Barriers and support for digital devices and Web 2.0 adoptions

Findings presented in this section emerged from the interview data. In particular, they respond to two of the interview questions which asked about the barriers that have been encountered by the participants in the adoption, and the support they would need.

Technologies are numerous and there are significant challenges associated with the selection of devices or Web2.0 tools. The challenges and barriers to technology adoption identified in this research included: time to consider and learn new technologies, trustworthiness of information, dispersed information, selection of technology, and reliability and interoperability of technology. In addition, there are often challenges around security with password management and backup of information across various devices. It was identified that technology is largely responsible for information overload which is a challenge for medical students and educators. If not used appropriately, technologies can be disruptive and to interfere with deep learning and effective and efficient professional practice. As it is discussed by an academic:

*… technologies actually being designed to not interfere with your life – things like the pop-up text that you’ve got a message … the default that you are instantly accessible … they detract from deep-thought processes and I think they are detrimental.*

Learning about new technologies, such as a digital device or a Web 2.0 tool, is a time-consuming activity. There is a need to develop a level of proficiency in the use of technologies for both students and educators. Students expect their lecturers to be proficient and to model good practice in technology use. Becoming proficient, however, requires that appropriate support and training is provided and time is available to those who desire to enhance their skills. Online help is considered to be useful, while skilled personal support at the point of need is also generally desired. Exemplars can be helpful to demonstrate utility and good practice in technology use for a particular purpose. Conversations around technology use are also believed to be beneficial, with recommendations and support from peers being highly valued in particular. The user interface of a technology was mentioned, with the ease of use and appealing presentations being emphasised.

## Discussion

Learners have the desire and need to be able to freely choose their preferred technologies [3, 25], and this project was designed to facilitate this choice. The research revealed that being able to access a range of tools through devices of their choice was highly desirable by medical students and educators. While computers remain as the dominant device by students and educators in medical education, a desire for mobile device adoption has been observed in this study. Utilising a specific device can be seen as an extension of the learners’ personality and learning style [26]. This finding reflects the overall increase in mobile device adoptions in the health sector and the transformative potential of these devices and available apps [11, 26, 27].

Regarding the Web 2.0 adoption, a mismatch was observed between the users’ desire and their actual practice. The participants in this research revealed a high level of desire and expectation for more efficient and effective learning and professional practice achieved through using Web 2.0 technologies. However, in practice, only less than half of the Web 2.0 tools listed are being frequently used by this participant cohort. This mismatch is reflected in other existing literature. On the one hand, Web 2.0 technologies that are readily available on the internet have significant capacity to enhance learning [3, 28]. On the other hand, while there is a potential for these tools to help facilitate collaborative, interactive and student-centred learning [29], there are other influential factors to determine users’ level of acceptance and adoption of new technologies. There are a multitude of purposes, as found in this study, for users’ adoption of Web 2.0 tools. Understanding these purposes and providing guidance in the selection of tools would lead to more effective adoption.

The findings in this study reflect the importance of training and support as a significant contributor to technology adoption [26, 30]. The participants reflected on a number of barriers that are associated with the adoption. To address these challenges, both pedagogical and technological support is paramount, and approaches will need rethinking to help learners “navigate this complex and interconnected landscape” [31]. Differences have been observed between the adoption of the students, who are also the younger age group, and the staff, who are also the older group. These differences are also reflected in previous research [6]. To address this higher level of interest from students, educators will need to equip themselves with the relevant knowledge and skills, which could be facilitated within the medical education courses or at an institutional level. In particular, for the digital habitat development and implementation at the second stage of this research, support will need to be provided to assist users in the selection of Web 2.0 tools and for effective adoption of these tools to fulfil their educational purposes.

## Conclusions

This paper reported the findings of Stage 1 of a two-staged study. While the whole study aimed to develop a personalised digital habitat for medical students and educators in the chosen organisation, the first stage of the study aimed to examine the types of devices and Web 2.0 tools that are being used by the potential users of this digital habitat. This first stage provided a user analysis for the second stage. One key message discussed in this paper is the desire for learners to be able to freely choose Web 2.0 technologies that support their individual needs and preferences. Currently, there is a mismatch between users’ desires and the actual practice. The paper also highlighted the role of organisations in facilitating the Web 2.0 adoption for educational purposes, and in providing relevant training to respond to the mismatch. The discussions and conclusions of this paper are relevant to medical education institutions, who recognise the changes brought by these technologies and prepare to respond to these changes. This paper provides the foreground information for a future paper which will discuss the finds of the second research stage, including the digiMe development, implementation and evaluation.

### Ethics approval and consent to participate

Ethics approval was obtained from the Tasmanian Social Sciences Human Research Ethics Committee (Reference Number H0012730).

### Consent for publication

Consent has been obtained from the participants for including their responses in publications.

### Availability of data and materials

The data supporting the findings of this paper is stored at the University of Tasmania. Please contact the authors if you wish to access the data.

## Additional files

Additional file 1: Questionnaire Part B, Part C and Part D. (DOCX 14 kb) Additional file 2: Interview questions. (DOC 22 kb)

## Abbreviations

LMSs: learning management systems
PASW: predictive analytics software
PDAs: personal digital assistants
PLEs: personalised virtual learning environments
