# Supplementary material for: A mixed-method research to investigate the adoption of mobile devices and Web2.0 technologies among medical students and educators
Source: BMC Med Inform Decis Mak. 2016 Apr 19;16:43. doi: 10.1186/s12911-016-0283-6 (PMC4837580; doi:10.1186/s12911-016-0283-6)
Supplement: Additional file 1: — Questionnaire Part B, Part C and Part D. (DOCX 14 kb) [file 12911_2016_283_MOESM1_ESM.docx]

**Additional file 1: Questionnaire Part B, Part C and Part D**

**Part B**

What devices? Please indicate the types of mobile devices that assist you with activities or management in your learning, teaching or professional day. You may select more than one.

iPhone

Android smartphone

Blackberry smartphone

Windows smartphone

iPad

Android tablet

Blackberry tablet

Windows tablet

No mobile devices used

Other(s) (Please specify) _______________

**Part C**

Which of the following technologies do you use to assist with your regular work/learning activities?

Directions: Please respond using the following criteria:

1 = Never

2 = Occasionally (less than once per week)

3 = Frequently (1 to 5 times per week)

4 = Very frequently (6 or more times per week)

| Types of Web 2.0 tools | 1 | 2 | 3 | 4 |
| --- | --- | --- | --- | --- |
| Instant messaging |  |  |  |  |
| Social networking tool (e.g. Yammer, Facebook) |  |  |  |  |
| Cloud storage (e.g. DropBox, iCloud) |  |  |  |  |
| Learning Management System (e.g. MyLO, Moodle) |  |  |  |  |
| Web authoring tool |  |  |  |  |
| Wiki |  |  |  |  |
| Blog |  |  |  |  |
| SmartBoard |  |  |  |  |
| eReader |  |  |  |  |
| Annotating tool (e.g. iAnnotate) |  |  |  |  |
| Social Bookmarking |  |  |  |  |
| Collaborative authoring tool (e.g. GoogleDocs) |  |  |  |  |
| Digital stylus for writing/drawing |  |  |  |  |
| Video capture tool |  |  |  |  |
| Video/slide viewing (e.g. YouTube) |  |  |  |  |
| Slide creation tool (e.g. PowerPoint, Slideshare) |  |  |  |  |
| Audio recording tool |  |  |  |  |
| Audio listening tools (podcasts) |  |  |  |  |
| Desktop capture (e.g. Echo 360, Camtasia) |  |  |  |  |
| Online chat (e.g. MSN) |  |  |  |  |
| Web conferencing (e.g. Elluminate) |  |  |  |  |
| VOIP internet tele- or video-conferencing (e.g. Skype) |  |  |  |  |

Other frequently used non-clinical technologies (please specify) _______________

**Part D**

How could technologies (digital devices and Web 2.0 tools) be used to improve your learning and teaching?

How could technologies (digital devices and Web 2.0 tools) be used to improve your clinical practice?

How could technologies (digital devices and Web 2.0 tools) be used to improve your professional practice?
