# Supplementary material for: A mixed-method research to investigate the adoption of mobile devices and Web2.0 technologies among medical students and educators
Source: BMC Med Inform Decis Mak. 2016 Apr 19;16:43. doi: 10.1186/s12911-016-0283-6 (PMC4837580; doi:10.1186/s12911-016-0283-6)
Supplement: Additional file 2: — Interview questions. (DOC 22 kb) [file 12911_2016_283_MOESM2_ESM.doc]

**Additional file 2: Interview questions**

1. What processes or activities do you perform in your role with the University of Tasmania that you find cumbersome, time-consuming or inefficient?
2. Would you like suggestions for technologies (digital devices and Web 2.0 tools) that might enable you to improve these processes or activities?
3. Are there any particular technologies (digital devices and Web 2.0 tools), or technology assisted processes, that you think might be able to help improve efficiency or effectiveness in your learning and teaching or other professional activities?
4. What would incline you to learning a new technology (digital devices or Web 2.0 tools)?
5. What would be a barrier to want to learn a new technology (digital devices or Web 2.0 tools)?
6. If training and ongoing support were available to learn technologies (digital devices and Web 2.0 tools) to improve efficiency or effectiveness in your learning and teaching or other professional activities, would you access them?
